# Relationships between serum levels of lactate dehydrogenase and neurological outcomes of patients who underwent targeted temperature management after out-of-hospital cardiac arrest

**DOI:** 10.1097/MD.0000000000026260

**Published:** 2021-06-18

**Authors:** Yeon Ho You, Yong Nam In, Jung Soo Park, Insool Yoo, Seung Whan Kim, Jinwoong Lee, Seung Ryu, Jin Hong Min, Won Joon Jeong, Yong Chul Cho, Se Kwang Oh, Hong Joon Ahn, Chang Shin Kang, Byung Kook Lee, Dong Hun Lee, Dong Hoon Lee, Gyeong Gyu Yu

**Affiliations:** a Department of Emergency Medicine, Chungnam National University Hospital; b Department of Emergency Medicine, College of Medicine, Chungnam National University, Jung-gu, Daejeon; c Department of Emergency Medicine, Chungnam National University Sejong Hospital, Sejong; d Department of Emergency Medicine, Chonnam National University School of Medicine; e Department of Emergency Medicine, Chonnam National University Hospital, Dong-gu, Gwangju; f Department of Emergency Medicine, College of Medicine, Chung-Ang University, Dongjak-gu, Seoul; g Department of Emergency Medical Service, Seojeong University, Eunhyeon-myeon, Yangju-si, Gyeonggi-do, Republic of Korea.

**Keywords:** cardiac arrest, lactate dehydrogenase, targeted temperature management

## Abstract

This study aimed to evaluate times for measuring serum lactate dehydrogenase levels (SLLs) to predict neurological prognosis among out-of-hospital cardiac arrest (OHCA) survivors.

This retrospective study examined patients who experienced OHCA treated with targeted temperature management (TTM). The SLLs were evaluated at the return of spontaneous circulation (ROSC) and at 24, 48, and 72 hours later. Neurological outcomes after 3 months were evaluated for relationships with the SLL measurement times.

A total of 95 comatose patients with OHCA were treated using TTM. Seventy three patients were considered eligible, including 31 patients (42%) who experienced good neurological outcomes. There were significant differences between the good and poor outcome groups at most time points (*P* < .001), except for ROSC (*P* = .06). The ROSC measurement had a lower area under the receiver operating characteristic curve (AUC: 0.631, 95% confidence interval [CI]: 0.502–0.761) than at 48 hours (AUC: 0.830, 95% CI: 0.736–0.924), at 24 hours (AUC: 0.786, 95% CI: 0.681–0.892), and at 72 hours (AUC: 0.821, 95% CI: 0.724–0.919).

A higher SLL seemingly predicted poor neurological outcomes, with good prognostic values at 48 hours and 72 hours. Prospective studies should be conducted to confirm these results.

## 1 Introduction

The survival rate for patients with out-of-hospital cardiac arrest (OHCA) has increased because of improved education regarding cardiopulmonary resuscitation, including the use of automated external defibrillators and the development of post-cardiac arrest syndrome (PCAS) therapy, such as targeted temperature management (TTM). However, despite optimal care and successful cardiopulmonary resuscitation, many patients are disabled or experience brain death.^[1]^ Hypoxic-ischemic brain injury(HIBI) is a major cause of disability, contributes to morbidity and mortality after OHCA, and may result in significant neurological sequelae.^[2,3]^ During TTM, physicians need to be aware of the neurological and survival outcomes of comatose patients. Early prognostic stratification of OHCA survivors is essential to tailor appropriate management plans and efficiently allocate resources.^[4,5]^ Thus, several studies have aimed to develop tools for predicting neurological and survival outcomes, although no single effective method has been found for predicting these outcomes.^[6,7]^ Several biological markers, such as the S-100B protein and neuron-specific enolase (NSE), have been demonstrated to be useful in this setting. Moreover, electroencephalography and neuroimaging, including brain computed tomography (CT) and magnetic resonance imaging (MRI), have been used by physicians to predict neurological outcomes.^[5–9]^ Nevertheless, a more effective method for predicting survival and neurological outcomes early in the treatment process would be useful for planning PCAS care.

Lactate dehydrogenase (LDH) is a cytoplasmic enzyme that is widely expressed in tissues.^[10,11]^ The enzyme converts pyruvate, which is the final product of glycolysis, to lactate when oxygen is in short supply, and it is detectable in the serum. The LDH enzyme exists in all cell types and tissues, including the muscles, liver, and brain, where it is released into the plasma by cellular damage,^[10,11]^ leading to abruptly increased serum LDH levels(SLLs) when organs are damaged during hypoxia.^[12–14]^ Our theory is that HIBI severe enough to affect the brain will co-occur with extensive tissue damage in 1 or several organs, which would make a non-organ-specific biochemical marker, such as total LDH, preferable. In this context, increased ischemic severity in patients with OHCA would presumably lead to increased SLLs. Recent studies have indicated that SLL may be an inexpensive and safe prognostic marker in patients with neonatal hypoxic-ischemic encephalopathy and traumatic brain injury. It may be correlated with neurological prognosis.^[12–18]^ However, the relationships between SLL changes and neurological and survival outcomes have not been studied in adult OHCA survivors. Furthermore, no study has examined the longitudinal changes in SLLs during the early PCAS interval. Thus, the present study evaluated whether serial changes in SLLs at the return of spontaneous circulation (ROSC), 24 hours later, 48 hours later, and 72 hours later would be correlated with neurological and survival outcomes among patients undergoing TTM.

## 2 Methods

### 2.1 Study design and population

This retrospective observational study evaluated non-traumatic adult OHCA survivors who underwent TTM at a Korean university hospital between January 2015 and August 2018. The study protocol was approved by the Institutional Review Board (2018-12-043). Patients were excluded if they

1. expired or were transferred within 72 hours after ROSC,
2. had experienced extracorporeal membrane oxygenation,
3. were diagnosed with cancer, or
4. had missing data.

### 2.2 TTM protocol

All comatose non-traumatic OHCA survivors were eligible for TTM following the previously established PCAS care protocol.^[19,20]^ TTM was started within 6 hours, using an ArticSun (Bard Medical, Louisville, CO) to obtain a target temperature of 33°C, which was maintained for 24 hours. After 24 hours at 33°C, patients were slowly rewarmed to 37°C at a rate of 0.25°C/h. Patients were sedated using midazolam and were paralyzed using cis-atracurium during TTM. Arterial blood levels, arterial pressure, mean arterial pressure, and urine output of SaO_2_ 94% to 96%, PaCO_2_ 35 to 45 mm Hg, ≥70 mm Hg, and ≥0.5 mL/kg/h were respectively maintained. Electroencephalography was performed if the patient's consciousness deterioration, involuntary movements, or seizure persisted. If there was evidence of electrographic or a clinical diagnosis of seizure, anti-epileptic medications were administered.

### 2.3 Measurement of SLL

Our post-OHCA intensive care unit protocol includes repeated SLL measurements at 6 hours intervals after ROSC at initial 24 hours and then every 24 hours. The SLL values at ROSC, 24 hours, 48 hours, and 72 hours later were selected.

### 2.4 Outcomes

The study outcomes were the relationships between the SLL values at each measurement (at ROSC, 24 hours, 48 hours, and 72 hours later) and the 3-month neurological outcomes. The patients’ neurological statuses were determined using hospital records or by directly calling the patient's caregiver. Neurological outcomes at 3 months after the OHCA were assessed using the Glasgow-Pittsburgh cerebral performance categories (CPC) scale. It defines the outcomes as CPC 1 (good performance), CPC 2 (moderate disability), CPC 3 (severe disability), CPC 4 (vegetative state), or CPC 5 (brain death or death). For the present study, good neurological outcomes were defined as CPC 1–2, and poor neurological outcomes were defined as CPC 3–5. Life-sustaining therapy was not abrogated based on current legal requirements.

### 2.5 Data analysis

Categorical variables were presented as frequencies and percentages; comparisons were performed using χ^2^ or Fisher exact tests, as appropriate. Continuous variables were presented as median values with interquartile range (IQR) values or means and standard deviations. The Mann–Whitney test was conducted for comparisons of LDH levels between neurologic outcome groups. For each time point, receiver operating characteristic curves were plotted, and corresponding areas under the curve (AUC) were determined to evaluate the predictive performance of SLLs on poor neurologic outcomes. A sensitivity analysis was performed using 2 different cut-off values for predicting poor neurologic outcome at 3 months post-OHCA based on a maximal Youden index (sensitivity + specificity – 1) and cut-off values with 100% specificity. Delong test was used to determine prognostic performance differences between poor neurologic outcome and each of the time points (at ROSC, 24 hours, 48 hours, and 72 hours after ROSC). Data were analyzed using SPSS software (version 18; SPSS Inc., Chicago, IL) and receiver operating characteristic curves were calculated and compared using MedCalc software (version 15.2.2; MedCalc, Mariakerke, Belgium). A two-sided significance level of 0.05 was used to indicate statistical significance.

## 3 Results

### 3.1 Patient demographics

**Figure 1:**
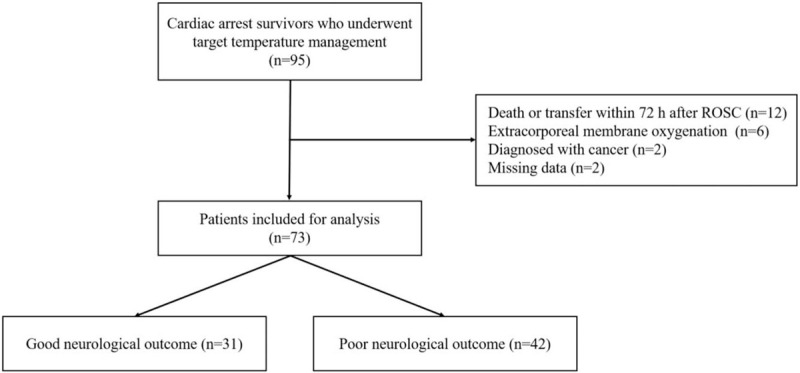
Study flow chart. ROSC: return of spontaneous circulation.

A total of 95 comatose OHCA survivors were treated using TTM, although 22 patients were excluded from this study. Thus, 73 patients were evaluated, including 31 patients (42%) who experienced good neurological outcomes (Fig. 1). The patients’ demographic and clinical characteristics are shown in Table 1. Significant differences between the good and poor neurological outcome groups were observed in terms of age, no flow time, low flow time, and the Sequential Organ Failure Assessment score.

**Table 1:** Baseline demographic and clinical characteristics.

	All patients (n = 73)	Good outcomes (n = 31)	Poor outcomes (n = 42)	*P*
Age, years	53.9 ± 17.7	48.3 ± 17.0	58.0 ± 17.3	.02
Male sex, n (%)	51 (69.9)	24 (77.4)	27 (64.3)	.30
Witnessed arrest, n (%)	45 (61.6)	24 (77.4)	21 (50.0)	.03
Bystander CPR, n (%)	44 (60.3)	23 (74.2)	21 (50.0)	.05
Shockable rhythm, n (%)	25 (34.2)	16 (51.6)	9 (21.4)	.01
Cardiac etiology, n (%)	25 (34.2)	14 (45.2)	11 (26.2)	.13
No flow time, min (IQR)	2.0 (1.0–9.0)	1.0 (0.0–2.0)	7.0 (1.0–13.0)	<.001
Low flow time, min (IQR)	20.0 (10.0–30.0)	12.0 (7.0–20.0)	26.0 (16.0–35.0)	<.001
SOFA score (IQR)	11.0 (9.0–12.0)	10.0 (8.0–11.0)	12.0 (10.0–13.0)	<.001
Serum LDH concentration, U/L (IQR)				
At ROSC	871.0 (577.0–1,183.0)	679.0 (494.0–1,099.0)	988.5 (607.0–1,289.0)	.06
24 h after ROSC	804.0 (556.0–1,212.0)	594.0 (475.5–787.0)	1,057.0 (666.0–1,461.0)	<.001
48 h after ROSC	596.0 (483.0–1,248.0)	496.0 (402.5–573.0)	1,056.0 (582.0–1,445.0)	<.001
72 h after ROSC	654.0 (442.0–1,087.0)	480.0 (399.5–635.0)	1,067.0 (630.0–1,398.0)	<.001

### 3.2 Associations of SLL with neurological outcomes

At ROSC, there was no significant difference in the SLLs between the good and poor outcome groups (679.0 g/dL [IQR: 494.0–1,099.0 g/dL] vs 988.5 g/dL [IQR: 607.0–1,289.0 g/dL]). However, significant differences in the SLLs were observed between the good and poor outcome groups at 24 hours, 48 hours, and 72 hours (Table 1).

### 3.3 Comparing the prognostic performances of each SLL measurement

**Figure 2:**
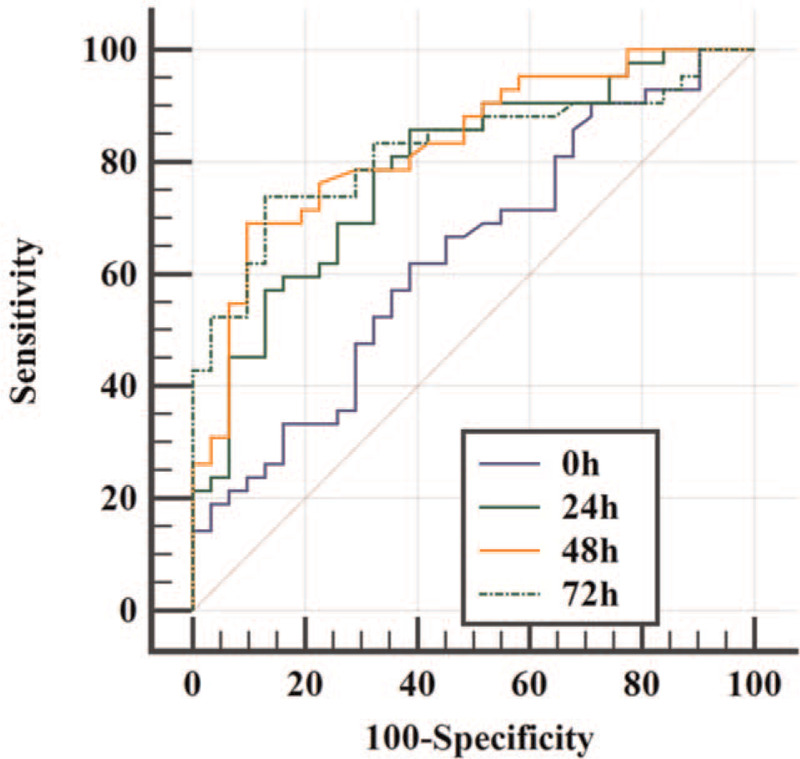
Comparing the receiver operating characteristic curves of serum lactate dehydrogenase levels at the return of spontaneous circulation (ROSC), 24 hours later, 48 hours later, and 72 hours later for predicting poor neurological outcomes.

The prognostic parameters for the SLL measurements at ROSC, 24 hours, 48 hours, and 72 hours later are shown in Table 2 and Figure 2. The AUC for the ROSC measurement was 0.631 (95% confidence interval [CI]: 0.502–0.761). The AUC measurement at 48 hours (0.830, 95% CI: 0.736–0.924) was higher than that of 24 hours (0.786, 95% CI: 0.681–0.892) and the measurement at 72 hours (0.821, 95% CI: 0.724–0.919). The cut-off value for the measurement at 48 hours was 698 g/dL, which provided 69.1% sensitivity and 90.3% specificity for predicting poor outcomes. Table 3 shows the prognostic parameters for the various SLL measurements based on 100% specificity, with the 72-hours SLL measurement having the best performance at 100% specificity (42.9% sensitivity, cut-off value: 1,087 mg/dL).

**Table 2:** Abilities of serum lactate dehydrogenase concentrations to predict neurological outcomes.

	AUC (95% CI)	*P*	Cut-off	Sensitivity/specificity	PPV/NPV
At ROSC	0.631 (0.502–0.761)	.06	807	61.9/61.3	68.4/54.3
24 h after ROSC	0.786 (0.681–0.892)	<.001	635	85.7/61.3	75.0/76.0
48 h after ROSC	0.830 (0.736–0.924)	<.001	698	69.1/90.3	90.6/68.3
72 h after ROSC	0.821 (0.724–0.919)	<.001	671	73.8/87.1	88.6/71.1

**Table 3:** Prognostic parameters for serum LDH at 100% specificity for predicting neurological outcomes.

	Cut-off	Sensitivity	Specificity
At ROSC	1,725	14.3	100
24 h after ROSC	1,671	21.43	100
48 h after ROSC	1,380	26.2	100
72 h after ROSC	1,087	42.9	100

## 4 Discussion

The development of HIBI after OHCA is a leading cause of long-term neurological disability and mortality,^[17]^ with the pathophysiology of HIBI involving a heterogeneous cascade that culminates in secondary brain injury and neuronal cell death.^[18]^ This begins with primary injury to the brain caused by the immediate post-arrest cessation of cerebral blood flow.^[18]^ The secondary injury of HIBI occurs during the hours and days following the OHCA and subsequent reperfusion. The factors implicated in this secondary injury include reperfusion injury, microcirculatory dysfunction, impaired cerebral autoregulation, hypoxemia, hyperoxia, hyperthermia, fluctuations in arterial carbon dioxide, and concomitant anemia.^[18]^ Rigorous studies have examined using TTM to prevent secondary injury after HIBI, and this treatment is associated with improved outcomes relative to hyperthermia.^[18–20]^ However, even when TTM is used, it is impossible to prevent all brain cell damage caused by the HIBI pathway, which can result in the release of various blood markers (e.g., LDH, NSE, and S100B).^[19,21–25]^

The LDH enzyme catalyzes pyruvate's reversible conversion to lactate during anaerobic glycolysis and is found extensively in the blood cells, brain cells, heart, muscle, and other body tissues.^[10–13]^ Cellular damage can release large quantities of LDH into the plasma, making LDH an important blood marker of cellular damage commonly used in HIBI experimental studies.^[10–15]^ Furthermore, LDH is chemically associated with medical conditions involving extensive tissue damage. Thus, we have hypothesized that HIBI severe enough to affect the brain will co-occur with extensive tissue damage in 1 or several organs, which would make a non-organ-specific biochemical marker (e.g., total LDH) preferable. Several studies have examined the prognostic value of LDH testing in infants.^[12–15]^ However, Beken et al reported that LDH only provided conclusive and statistically significant results if it was used as a component to determine the HIBI stage, which suggests that the related parameters only have predictive value if they are measured and interpreted together.^[26]^

It is important to predict outcomes before starting TTM; multiple clinical, laboratory, and imaging parameters have been used to predict outcomes among post-OHCA patients.^[5–9,19,26]^ However, there is no single definitive method that has been proven to be successful in this setting. Thus, identifying factors or combinations of factors that affect neurological outcomes could help guide new treatment regimens, plan rehabilitation programs, and counsel patients and families. In the context of predicting post-OHCA neurological outcomes, the available methods include prognostic tools (e.g., electroencephalography and N20 SSEP), blood markers (e.g., NSE and S100B), imaging (e.g., CT and MRI), and other clinical variables (e.g., pupil reflex and motor movement presence of myoclonus).^[5–9,19,26]^ Investigators have also attempted to assess the degree of HIBI using various blood markers, such as NSE, S100B, Tau, and lactate.^[7–9,26]^ However, only NSE has been accepted as being effective in this setting. When combined with other prognostic tests after CA, it may be reasonable to predict poor neurological outcomes based on high serum NSE values at 48 hours or 72 hours postarrest, especially if repeated sampling reveals persistently high values. However, the possibility of false-positive results indicates that serum NSE should not be used alone to predict poor neurological outcomes.^[7–9,19,26]^

Numerous studies have examined the relationship between LDH levels and head trauma prognosis. For example, Thomas et al^[11]^ studied LDH isoenzymes following head injury. In addition, Rao et al^[10]^ studied the relationship between SLLs and outcomes up to 15 days among 110 head trauma patients. Interestingly, Rao et al^[10]^ found that the measured SLLs were proportional to neurological injury severity. Patients who were unconscious for <1 hour had elevated SLLs only on the first post-injury day, while patients with longer periods of unconsciousness had prolonged SLL elevation, and patients who died or had severe disability had even more elevated SLLs. Furthermore, the lowest SLLs were observed among patients who were alive and without a disability, highlighting the strong relationship between SLLs and head injury severity.^[10,11]^ Jain et al also reported a significant relationship between SLL and outcomes after isolated traumatic brain injury, as elevated SLLs were observed in patients with poor outcomes, with continuously increasing SLLs observed during the study period for patients who developed disability or died.^[27]^ Neurological outcomes have also been predicted in infants using SLLs, as Yum et al and Thoresen et al found that SLL changes during the first 3 days after birth were associated with hypoxic-ischemic lesions in the central gray matter of infants with HIBI.^[13,15]^

The present study examined whether SLL elevation could predict severe HIBI, and the results indicate that elevations at the 48-h and 72-h measurements were significantly associated with poor neurological outcomes. Previous studies have indicated that NSE has high sensitivity at 100% specificity in this setting, although LDH testing is more widely available and less expensive.^[28]^ However, LDH should not be considered when assessing poor outcomes with a single test. Rather, it may be used as a component of the multimodal prognostication methods used to predict neurologic outcomes in OHCA survivors. We hope to develop a predictive model using a combination of clinical, electrophysiological, neurological, and biochemical indices of HIE. Incorporating SLL may help improve the prognostication and use of TTM for patients with altered biochemistry following OHCA.

The present study has several limitations. First, the small sample size and retrospective single-center design may limit the findings’ generalizability, especially as we only included patients with correctly recorded values at ROSC, 24 hours later, 48 hours later, and 72 hours later. Second, a large proportion of the evaluated patients was excluded because of missing data. This might have caused selection bias, which could also limit the generalizability of our findings. Third, significant differences were observed in the baseline characteristics of the good and poor outcome groups (e.g., age, witnessed arrest, and shockable rhythm). Furthermore, unclear data were excluded because of the inherent limitations of retrospective studies. Fourth, the present study excluded cases of death or transfer within 72 hours, as SLL measurements could not be completed in those cases. While these cases can be appropriately excluded from the analysis of post-death time points, they should likely not be excluded from pre-death time point analysis, which might have influenced our findings. Fourth, although we administered a specific amount of human albumin based on national insurance standards, this treatment might have influenced the relationship between neurological outcomes and SLL.

## 5 Conclusion

The present study revealed that elevated SLLs after ROSC, especially at 48 hours and 72 hours, could help predict poor neurological outcomes after TTM for OHCA. However, these findings require further validation in prospective studies, and LDH classification based on isoenzymes may help further enhance its prognostic value. Furthermore, prognostication could be improved by combining SLL with other variables, such as age, the initial Glasgow coma scale score, and CT and MRI findings.

## Author contributions

**Conceptualization:** Yeon Ho You, Yong Nam In, Jung Soo Park, Wonjoon Jeong.

**Data curation:** Yeon Ho You, Jung Soo Park, Jin Hong Min.

**Formal analysis:** Yeon Ho You, Jung Soo Park, Wonjoon Jeong.

**Investigation:** Jinwoong Lee, Hong Joon Ahn.

**Methodology:** Yong Nam In, Yong Chul Cho, Se Kwang Oh.

**Project administration:** Seung Ryu, Se Kwang Oh, Hong Joon Ahn.

**Resources:** Seung Whan Kim, Se Kwang Oh, Byung Kook Lee.

**Software:** Byung Kook Lee.

**Supervision:** Insool Yoo, Changshin Kang.

**Validation:** Dong Hun Lee, Gyeong Gyu Yu.

**Visualization:** Dong Hoon Lee.

**Writing – original draft:** Jin Hong Min.

**Writing – review & editing:** Yong Nam In.
